# CKD in reproductive-aged women: a call for early nephrology referral and multidisciplinary care

**DOI:** 10.1186/s12882-024-03864-9

**Published:** 2024-12-03

**Authors:** Nityasree Srialluri, Sumeska Thavarajah

**Affiliations:** Division of Nephrology, Johns Hopkins School of Medicine, Baltimore, MD USA

**Keywords:** Pregnancy, Chronic kidney disease, Early referral, Reproductive age women

## Abstract

Chronic Kidney Disease (CKD) affects millions globally, with a notable impact on biological females of reproductive age. This population faces specific issues such as fertility concerns, complex contraceptive decisions, and complications related to pregnancy that can exacerbate CKD. Given the increasing prevalence of CKD among young men and women owing to rising rates of hypertension, obesity, and diabetes, there is a need for early and tailored interventions among women of childbearing age. Current Kidney Disease Improving Global Outcomes (KDIGO) guidelines suggest nephrology referral primarily for advanced CKD stages or significant proteinuria. However, women at any CKD stage may face complex pregnancy-related decisions and increased risks that are not adequately addressed by these guidelines, warranting early specialty care. This review explores the distinct needs of women of reproductive age with CKD, identifies gaps in the existing management framework, and advocates for earlier and more comprehensive nephrology involvement. By focusing on preconception planning, risk factor management, adverse pregnancy outcomes, and existing disparities in care, this review seeks to improve understanding of the needs of women of reproductive age with CKD and calls for a shift towards more proactive, nephrology-driven care.

## Introduction

Chronic Kidney Disease (CKD) is a pressing global health issue, affecting an estimated 850 million people worldwide [1]. While CKD affects individuals across all genders and age groups, it has particularly profound impacts on biological females of reproductive age (referred to as women or females in this review), who face unique challenges. For these individuals, CKD complicates several aspects of health and family planning, including fertility, contraception choices, adverse pregnancy outcomes, and CKD progression. Unique health conditions, such as acute kidney injury (AKI) during pregnancy or hypertensive disorders of pregnancy, can significantly affect long term kidney health if not managed early. As rates of hypertension, obesity, and diabetes increase, the incidence of CKD among reproductive age women is expected to rise, highlighting the urgent need for early intervention and specialized care to improve outcomes [1]. This calls for a multidisciplinary approach to detect, monitor, and manage CKD in reproductive-age women.

Primary Care Providers (PCPs) play a crucial role in the detection and early management of CKD. Current Kidney Disease Improving Global Outcomes (KDIGO) guidelines recommend nephrology referral primarily for CKD Stage 3b or above or in cases with substantial proteinuria [2]. However, growing evidence suggests that biological females of reproductive age across all stages of CKD face increased risks that impact both kidney and reproductive health [3–8]. Women with resolved kidney injury, glomerulonephritis in remission, diabetes, or chronic hypertension often face increased risks of both kidney and reproductive complications [9–12]. Thus, current guidelines and risk prediction equations may not adequately reflect the needs of reproductive individuals with CKD.

PCPs, although crucial in CKD management, may not always have the resources or time to discuss and adjust treatment plans comprehensively, particularly for reproductive-age women who may require specialized counseling on safe medication use, management of proteinuria, and blood pressure during pregnancy. The current approach to the timing of nephrology referral may inadvertently place these women at higher risk by potentially delaying specialist intervention until their kidney disease has significantly progressed. A multidisciplinary approach that prioritizes early nephrology referrals can help mitigate disease progression and support safer pregnancies. This review aims to highlight the need for a shift in the approach to the management of CKD among biological females of reproductive age. We will discuss the unique needs of this population, review the current state of care and knowledge gaps, and offer recommendations for improving care and outcomes.

### Epidemiology, risk factors, and care disparities among women of reproductive age

Globally, CKD is more prevalent in women than men, and women younger than 45 have higher all-cause and non-cardiovascular mortality compared to men in the same age group [13, 14]. This increased mortality reproductive-age females is not fully understood, though it may be linked to the impact of CKD on conditions related to reproduction. Current estimates suggest that CKD affects up 6% of women of childbearing age in high-income countries and around 3% of pregnancies are impacted by CKD, although these figures likely underrepresent the true prevalence due to challenges in CKD diagnosis during pregnancy [5, 15, 16].

Diagnosis of CKD in pregnancy remains challenging due to inconsistencies in CKD definitions, single laboratory measurements, non-validated estimated glomerular filtration rates (eGFRs), and insufficient proteinuria data. A large Canadian population study reported a higher CKD rate based on pre-pregnancy eGFR, with 7.5% of pregnancies with mild CKD (eGFR 60–90 ml/min/1.73m^2^) [8]. However, two-thirds of participants were excluded due to missing or invalid baseline creatinine measures, underscoring the need for improved screening and diagnosis of CKD [8]. As the prevalence of CKD rises, so does the proportion of women of childbearing age with CKD, attributable to increasing rates of risk factors such as obesity, hypertension, and diabetes. Yet data from the 2023 USRDS annual report indicate that only 19.1% of patients aged 18–39 with CKD Stage 3 are receiving nephrology care, a significant portion of whom are women [17]. Notably, this statistic overlooks earlier stages of CKD, where reproductive consequences may be less prominent but still significant.

Care disparities in CKD disproportionately affect women, complicating timely diagnosis and management. Data from the United States National Health and Nutritional Examination Survey (NHANES) indicate that CKD awareness is approximately 10.2% lower in women aged 20–49 than in men, which is concerning given the significant implications of CKD on maternal and fetal health [18]. Additional recent studies highlight that women are less likely to be referred to nephrologists and often receive less intensive CKD management than men [19, 20]. A recent Stockholm study demonstrated that women with CKD are less frequently diagnosed, monitored, referred to nephrologists, and prescribed antiproteinuric medications compared to men [19]. Among individuals with diabetes and hypertension, women undergo fewer albuminuria measurements than men, and even when meeting referral criteria, they are less likely to visit a nephrologist within 18 months [19]. Men are also more likely to be referred to a nephrologist at higher eGFR and receive a CKD diagnosis sooner than women [21]. Women are also less likely to start Renin–angiotensin–aldosterone system inhibitors (RAASi) and are more prone to receive potentially inappropriate nephrotoxic medications [19, 22]. These disparities may be influenced by diagnostic limitations, as the use of serum creatinine rather than eGFR for CKD diagnosis can prevent early detection in women, whose lower baseline serum creatinine levels may obscure early signs of CKD. Additionally, prescriber caution regarding the use of RAAS inhibitors and other anti-proteinuric agents such as sodium-glucose cotransporter 2 inhibitors (SGLT2i) in reproductive-age women due to concerns about teratogenicity may also be contributing. Sociocultural factors may add further complexity as women’s prioritization of prioritize family health over personal health can lead to neglected CKD management [22]. These disparities are particularly pronounced in low socioeconomic areas and lower-middle-income countries, where access to comprehensive diagnostic tools, education, and regular monitoring is often limited [1, 23–25].

Pregnancy introduces further challenges in the diagnosis and management of CKD. Physiological changes during pregnancy, including fluctuations in glomerular filtration rate and proteinuria levels, can complicate accurate diagnosis and monitoring of CKD during this period [25]. Current eGFR equations may also underestimate CKD severity in pregnant individuals, creating potential barriers to appropriate care [25]. This challenge is compounded by pregnancy specific complications, such as hypertensive disorders of pregnancy which may not only exacerbate underlying CKD but can also lead to acute kidney injury (AKI) during pregnancy, with long-term ramifications for kidney health [26–31]. Individuals with CKD face a greater risk for AKI due to pregnancy complications such as hemorrhage, hyperemesis gravidarum, sepsis, thrombotic microangiopathies, autoimmune disease flares, and obstructive uropathy [31, 32]. Moreover, gestational diabetes or severe metabolic dysfunction during pregnancy can further increase the risk of CKD progression among women with CKD [11]. In high-income countries. advanced maternal age(women over 35 years old) giving birth has become more common and is associated with a range of adverse pregnancy outcomes, including miscarriage, pre-eclampsia, and gestational diabetes [33]. Though the risks may be small in magnitude, they can be compounded among women with CKD, who often have multiple other risk factors for adverse pregnancy outcomes. Recognizing and addressing these risk factors with targeted, pregnancy specific interventions, especially through early nephrology care, is essential to improve kidney outcomes in this vulnerable population.

### Pregnancy and CKD: risks and complications

Pregnancy poses significant challenges for woman with kidney disease due to the complex bidirectional interactions between kidney disease and pregnancy. Women with CKD are at risk for adverse pregnancy outcomes, which include AKI, worsening of proteinuria, and progression of underlying CKD [5, 6, 25]. They are also at increased risk of hypertensive disorders of pregnancy, particularly preeclampsia, which is associated with AKI, accelerated CKD progression, and end-stage kidney disease [26–30]. Recent data from United Kingdom reveal that 46% of pregnancies in women with CKD stages 3–5 had kidney disease progression -defined as at least 25% reduction in eGFR or the need for renal replacement therapy – within one year postpartum [34]. Additionally, pregnancies in women with CKD stages 3–5 has been shown to shorten the time to renal replacement therapy by 2.5–4.7 years [34].

Given these risks, individuals with advanced CKD should be counseled about the potential irreversible loss of kidney function during pregnancy, which can be severe enough to necessitate the initiation of dialysis. In addition to maternal risks, there is also a significant increase in adverse fetal outcomes such as small gestational age, neonatal intensive care unit admissions, intrauterine growth restriction, and even fetal demise [6, 7, 10, 16]. Maternal and fetal risks vary considerably across CKD stages and are exacerbated by the presence of comorbidities. Studies show that maternal and fetal risks are present even in earlier CKD stages, though generally to a lesser extent than in advanced stages [4–6, 35, 36]. For instance, worsening hypertension, increased proteinuria, and preeclampsia can develop in up to one-third of pregnant women with mild CKD [35, 37]. Prematurity (birth before 37 weeks), low birth weight, and fetal demise occur at slightly higher rates in women with mild CKD compared those without kidney disease [4, 6, 10, 34].

Comorbidities further elevate these risks, particularly diabetes, chronic hypertension, and autoimmune disorders which can significantly affect maternal and fetal outcomes if not well controlled before pregnancy [10–12, 38, 39]. Pregnancy-related AKI has a significant impact on maternal and fetal outcomes [9, 31]. During the postpartum period, hemorrhage, infections, antibiotics, and nonsteroidal anti-inflammatory drugs, can all increase the risk of AKI [32].

To manage these risks effectively, patients require thorough education on the potential complications and need for close monitoring and physicians’ guidance in selecting the safest timing for pregnancy. Given the elevated risk of adverse pregnancy outcomes (APOs) across all CKD stages, a multidisciplinary approach that includes early involvement of a nephrologist is essential to optimize outcomes for both mother and child.

### Pregnancy planning among biological females of reproductive age

Pregnancy planning is a critical aspect of care for women with CKD of reproductive age, given the significant impact of kidney disease on fertility and overall reproductive health. CKD often leads to sexual dysfunction and decreased fertility, stemming from both hormonal imbalances and physiological changes. While the underlying causes are only partially understood, they include reduced libido, dyspareunia, and disruptions in the hypothalamic gonadal axis [40–44]. The specific effects of CKD on the axis include impaired ovulation (menstrual cycle disruptions, anovulation, and hypoestrogenism), dysfunctional uterine bleeding, hyperprolactinemia (increased production and reduced clearance in CKD), and menopause [40, 43, 45]. Studies show that approximately 80% of women with CKD report sexual dysfunction, and up to 40% experience menstrual abnormalities [42, 46–48]. The degree of impairment in the hypothalamic gonadal axis is correlated with the severity of the CKD stage, emphasizing the importance of family planning at earlier stages of CKD [41].

For women with advanced CKD, the timing of conception is a critical factor influencing fertility. Fertility rates are notably higher in those who conceive before dialysis initiation, likely due to the hormonal and physiological disruptions associated with dialysis treatment [49]. Additionally, the reproductive lifespan of women with CKD has been found to be approximately 32 years, significantly shorter than the general population’s average of 37 years [47]. This shortened reproductive lifespan is a critical sex-specific factor that is associated with a higher future kidney and cardiovascular risk [50–52]. Sexual dysfunction in women with CKD also impacts psychosocial health, contributing to anxiety, loss of self-confidence, and depression and has long term impacts on cardiovascular disease and mineral bone disorder [42, 53]. Therefore, nephrologists must understand the pathophysiology, clinical manifestations, and treatment of sexual dysfunction, collaborating closely with obstetric gynecologists to enhance awareness and improve the quality of life for these patients.

Contraceptive counseling is an important aspect of care for this population. Women with kidney disease have risk factors such as hypertension, diabetes, and thromboembolic disease that require careful consideration of the choice of contraceptive use due to the inherent risk of blood clots and hypertension with some contraceptives. Despite the complexity surrounding pregnancy planning among women with CKD, proactive reproductive health discussions-including contraception counseling- are often overlooked. Less than a third of nephrologists report discussing menstrual irregularities and fertility with their patients, despite half acknowledging that their female patients desire these discussions [54–58]. Women with CKD contemplating pregnancy report frequently feeling ill-equipped to make informed decisions about pregnancy, often due to limited guidance from their healthcare providers about the impacts of CKD on reproductive health [54, 59–61]. Patients report increased confidence to proceed with a pregnancy when supported by their nephrologist and when care is coordinated with their primary care providers and obstetricians [56]. Even patients with mild CKD have expressed feelings of loss of autonomy or significant fears related to pregnancy, similar to those with advanced CKD, highlighting the need for proactive discussions at all CKD stages [55].

For some women with impaired fertility and sexual dysfunction, assisted reproductive technologies (ART) may be necessary to achieve pregnancy. The risks associated with ART in CKD are not fully understood at this time due to limited data. In vitro fertilization (IVF) treatments in women with CKD carry the risk of ovarian hyperstimulation syndrome (OHSS), a potentially life-threatening complication that can lead to massive fluid shifts and AKI [62]. Studies report that 7.4% of women with CKD undergoing IVF develop OHSS, a higher rate than in the general IVF population [63, 64]. Severe OHSS in CKD patients can cause AKI through hypovolemia, ureteric obstruction due to ovarian enlargement, or ischemic acute tubular necrosis [63, 65]. Additionally, IVF increases the likelihood of multifetal pregnancies, which independently elevates the risk of adverse pregnancy outcomes in women with CKD [64, 66]. Therefore, early and comprehensive family planning discussions are essential for managing pregnancy-related risks and improving long-term outcomes in women with CKD. Proactive reproductive counseling and coordinated care can empower women make informed decisions as they navigate the complexities of CKD, fertility, and pregnancy.

### Current practices and guidelines for management of CKD in reproductive age women

The management of CKD in reproductive-age women presents unique challenges, especially in areas of reproductive health, contraception counseling, and medication management. Current practices often do not fully address the complex needs of this population, leading to missed opportunities for timely intervention and comprehensive family planning.

The KDIGO 2024 guidelines recommend referring adults with CKD to specialist kidney care in cases of advanced CKD (eGFR < 60), rapidly declining kidney function, significant albuminuria (> 300mg/g), refractory hypertension, or need for renal replacement therapy [2]. Additional guidance suggests referral for patients with 3–5% risk according to validated risk tool, an absolute GFR < 30, or a urine albumin creatinine ratio > 300mg/g [2]. While KDIGO personalized approaches consider age, sex, and gender, there are currently no specific recommendations for young women of reproductive age who may benefit from early nephrology consultation. Current risk prediction models, focused on identifying kidney failure risk over 2–5 years in patients with eGFR < 60 ml/min/m2, are less effective for early CKD stages and do not account for the impact of pregnancy on CKD progression. The American Heart Association has recognized hypertension in pregnancy as a risk factor for future cardiovascular disease and stroke [65]. However, clinical guidelines have not yet addressed the role of reproductive risks in future kidney health. A history of pregnancy outcomes and complications in women with kidney disease should be collected systematically by all nephrologists to increase our understanding of the interplay of kidney health and pregnancy and to inform future guidelines.

Contraception counseling is crucial for preventing unintended pregnancies in women with CKD, as the risks of maternal and fetal complications increase when kidney disease and related comorbidities are poorly controlled. Despite recommendations, contraception use remains low in women with kidney disease, and few nephrologists discuss fertility and contraception with their patients [54, 58–60, 67]. Many women report frustration with their lack of knowledge about reproductive health, delays in receiving information, and lack of discussions regarding contraception [56, 68]. Nephrologists often report low confidence in initiating and supporting these conversations, citing limited training and time constraints, which leads to missed opportunities for early and safe pregnancy planning [54, 55, 57].

Patients with advanced CKD especially require intensive counseling, coordination of care, and individualized management. Despite reduced fertility in advanced CKD, conception remains possible at all stages of CKD. Many patients with advanced CKD are incorrectly advised that they are infertile, leading to an increased risk of unplanned and high-risk pregnancies [55]. Due to improved fertility and outcomes with kidney transplantation compared to advanced CKD and dialysis, women are often advised to delay conception for 1–2 years post-transplantation and be informed about the benefits and risks of immunosuppressive agents [15, 69–71]. However, with transplant wait times often extending 5–10 years, women may face delayed conception into advanced maternal age, which is associated with risks for both mother and fetus.

Effective pre-pregnancy counseling should also include screening for fetotoxic medications, maintaining well-controlled blood pressure, and establishment of timeline for close monitoring. Guideline-recommended anti-proteinuric agents such SGLT2i, RAASi, Mineralocorticoid Receptor Antagonists (MRAs), and Glucagon-like peptide-1 (GLP1) agonists are all contraindicated during pregnancy due to teratogenicity, yet guidelines lack specific timing recommendations for discontinuation and reinitiation post pregnancy [72, 73]. This lack of guidance in combination with inadequate counseling on contraceptive use in individuals of childbearing age who are prescribed these teratogenic medications poses a significant risk. Without proper contraceptive planning, there is an increased risk of unplanned pregnancies, potentially leading to adverse fetal outcomes. Standardizing pre-pregnancy counseling on medication safety and providing accessible resources on medication risks with early involvement of nephrologists could help mitigate these issues.

Family planning for women with CKD is complex, as sociocultural pressures and personal desires to conceive may conflict with concerns about birth abnormalities, serious medical risks and perceived burden on family [42]. A well-coordinated multidisciplinary team – including nephrologists, PCPs, and obstetricians—is essential to support informed decisions, reduce unplanned pregnancies, and provide comprehensive prenatal care. Nephrologists can focus on CKD progression and medication adjustments, obstetricians on pregnancy-specific risks, and primary care providers on broader contraceptive and health education, creating a supportive network for optimal patient outcomes.

### Recommendations for care and importance of early referral

Managing CKD in reproductive-age women requires a proactive, multidisciplinary approach to reduce the risks associated with pregnancy. Early nephrology referral, comprehensive family planning, and personalized reproductive counseling are essential to ensure optimal outcomes (Tables 1 and 2).

**Table 1:**
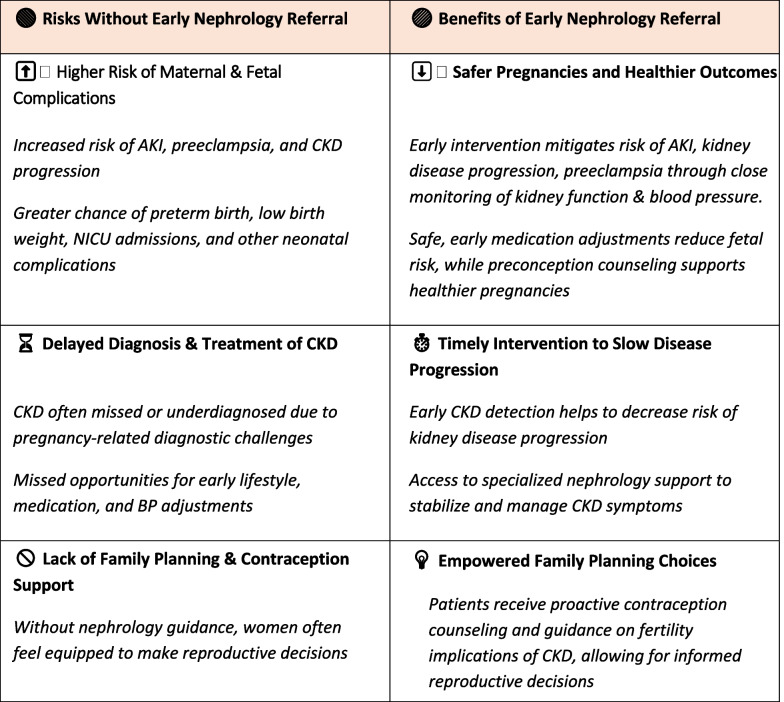
Early nephrology referral: essential for protecting reproductive health in women with CKD

**Table 2:**
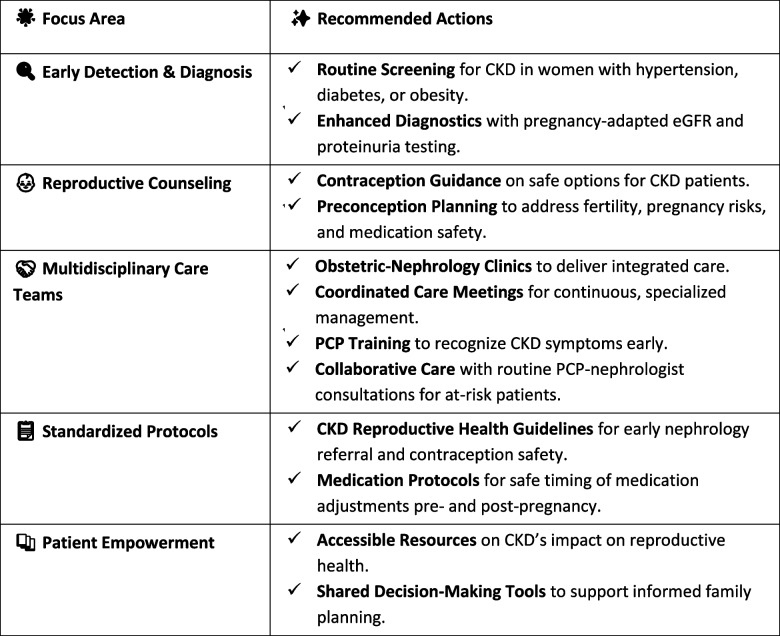
Key strategies for improved CKD care in reproductive-age women

#### Early nephrology referral and monitoring

Timely referral to nephrology is critical, as pregnancy-related risks in CKD patients are considerably lower in those with well-preserved kidney function, minimal proteinuria, controlled blood pressure, and underlying disease remission. Early nephrology involvement enables close monitoring of kidney function, blood pressure, and proteinuria, which are key indicators of potential pregnancy complications. By addressing these modifiable risk factors, such as hypertension, diabetes, obesity, and proteinuria, providers can help mitigate disease progression and improve maternal and fetal outcomes.

PCPs play a fundamental role in early identification of CKD and in initiation of discussions about reproductive health. PCPs are in a position to detect early CKD sin reproductive-age women and making them vital in setting the stage for early nephrology referral PCPs should work collaboratively with nephrologists, who can provide specialized guidance and tailor care based on CKD stage and individual comorbidities.

While specific recommendations among women of reproductive age are scarce, early referral to nephrology among all patients has been shown to improve long-term outcomes, mainly when interventions to prevent disease progression are initiated at higher eGFR [74, 75]. Early nephrology involvement is especially important for pre-pregnancy planning and ongoing monitoring, as some treatments are contraindicated during pregnancy. Nephrology engagement supports comprehensive care, facilitating medication review, kidney function assessment, and discussions on the impact of CKD on fertility and pregnancy. Close nephrology monitoring throughout pregnancy and postpartum ensures timely re-initiation of disease-modifying medications post-delivery, guided by individual risk assessments. This proactive approach helps patients make informed family planning decisions and mitigates the risk of adverse pregnancy outcomes.

The increasing incidence of kidney disease, coupled with a shortage of nephrologists, prevents all patients with CKD from being seen by a specialist. The outcomes for biological females of reproductive are generally favorable for the early stages of CKD compared to advanced CKD. We recognize that overtreatment can lead to unnecessary stress and interventions. However, given the complexity of family planning, the young age of the population, and the availability of an arsenal of kidney protective agents, the earlier stages of CKD present a larger opportunity to mitigate pregnancy-related adverse outcomes and CKD progression.

#### Individualized family planning and reproductive counseling for women with CKD

Family planning should begin early in the management of women with CKD. Comprehensive history taking including obstetric and reproductive history, is crucial for accurate risk assessment to inform potential pregnancy complications and CKD progression. This assessment lays the groundwork for initiating individualized discussions about contraception and pregnancy planning. Discussions should cover the impact of CKD on fertility, noting that while fertility decreases as CKD progresses, pregnancy remains possible even at advanced CKD stages. Patients should be informed about the heightened maternal and fetal risks, including the significant risk of pregnancy association progression of kidney disease and the possibility of dialysis initiation so that they can make well-informed decisions. Additionally, counseling should address the possibility of improved fertility and pregnancy outcomes following kidney transplantation [15, 70, 71].

Contraceptive options should be thoroughly discussed, with education about the risks and benefits of various methods in the context of CKD. These discussions should be provided by both PCPs and nephrologists, ideally in coordination with obstetrician-gynecology. For best outcomes, these discussions start at early stages of CKD, before disease progression limits reproductive choices.

Women with CKD require close monitoring and optimization of blood pressure and kidney function before planning a pregnancy, regardless of their disease stage. Assessing kidney function during pregnancy is challenging due to physiological changes like increased hyperfiltration, renal blood flow, and volume distribution, which can complicate CKD staging. In this scenario, frequent lab work, and a 24-h urine collection may be necessary for eGFR assessment, making regular visits with a nephrologist trained in these nuances essential for high quality care.

Medication management during pregnancy is crucial, as discontinuing fetotoxic medications, such as antiproteinuric agents, carries a risk of CKD progression and requires close nephrology oversight. Counseling should provide clear guidance on timing of medication discontinuation and include monitoring plans for kidney function and proteinuria. Current guidelines recommend avoiding anti-proteinuric agents in pregnant women or those planning a pregnancy. For patients on ACEi, discontinuation is generally advised once conception is planned or when pregnancy is confirmed in patients with high-risk proteinuric kidney disease [69]. Due to limited data on first-trimester exposure to SGLT2i, MRAs, ARB, and GLP1 agonists, and some reports suggesting potential fetal risks, these medications should also be discontinued during conception planning and avoided during pregnancy [69, 72].

Blood pressure management in pregnancy requires careful balancing to optimize maternal and fetal outcomes. Aggressive treatment should be avoided to reduce the risk of decreased uteroplacental flow and potential growth restriction. Although current recommended blood pressure threshold for antihypertensive initiation is 140/90, the optimal blood pressure target for pregnancy remains unknown [76]. Given the increased risk of preeclampsia in CKD, low-dose aspirin is recommended for all women with CKD between 12 and 28 weeks of gestation [77]. Differentiating preeclampsia from CKD progression can be challenges due to overlapping pathophysiology and symptoms. Rapid increases in proteinuria can occur due to withdrawal of antiproteinuric medications and pregnancy related hyperfiltration, complicating diagnosis. Specific markers, such as elevated liver function tests, thrombocytopenia, FDA-approved ratio of soluble Fms-like tyrosine kinase 1 (s-Flt-1) over placental growth factor (PIGF) can aid in distinguishing preeclampsia from CKD progression [25, 78].

### Proposed strategies for optimizing care

#### Establishing multidisciplinary team for comprehensive care

Multidisciplinary teams (MDT) composed of nephrologists, obstetricians, PCPs, and other specialists is essential to provide comprehensive care for women of reproductive age with CKD and improve outcomes. MDTs ensures comprehensive care is delivered by integrating reproductive health and CKD management, developing personalized care strategies, and leveraging diverse expertise to manage complex cases. They facilitate early detection and intervention, offer education and counseling on reproductive health, and address the specific needs of women with CKD, aligning with their desire for individualized, multidisciplinary care. Establishing dedicated obstetric nephrology clinics provides an environment where nephrologists can closely collaborate with obstetricians to manage the care of women with CKD. In these clinics, teams can provide continuous care, coordinate medication management, and address complications arising during preconception, pregnancy, and postpartum. This setup supports quality care, enhances expertise in complex CKD cases, and strengthens communication between specialists.

#### Enhanced training and education for primary care and nephrologists

Specialized training for nephrologists on reproductive health in CKD patients will improve the quality of reproductive counseling and family planning. Nephrology fellowship programs and continuing medical education should include modules on topics such as fertility counseling, pregnancy-related CKD risks, contraceptive options, and safe medication practices. Training in these areas will equip nephrologists in proactive discussions y to address the specific reproductive health needs of women with CKD confidently. Efforts should be made to educate PCPs on the impact of CKD on fertility, pregnancy outcomes, and the importance of early referral. PCPs should understand the basic pathophysiology and progression of CKD, as well as the associated reproductive risks across CKD stages. They should be prepared to initiate early and ongoing contraceptive counseling and recognize when timely referrals to obstetrics and nephrology are needed, especially for patients planning to conceive or at risk of unplanned pregnancies.

#### Developing clear guidelines for reproductive health in CKD

Standardized, evidence-based guidelines on reproductive counseling, family planning, and pregnancy management for CKD patients would help improve care consistency and provider confidence. Clear protocols are needed for contraceptive counseling, timing for discontinuing teratogenic medications, and risk management during pregnancy. Additionally, guidelines should address early nephrology referral criteria for young women with CKD, especially those planning pregnancy or requiring specialized reproductive health support. These tailored protocols would provide a structured framework for PCPs, nephrologists, and OB-GYNs to deliver coordinated and consistent care across settings. Examples for early referral guidelines exist internationally; for instance the National Institute for Health and Care Excellence(NICE) Guidelines for CKD recommend referral for young people to a specialist with any decrease of eGFR or a persistent ACR of 3mg/mmol or more [79]. These guidelines could serve as a valuable model for developing similar standards for childbearing women with CKD.

## Conclusion

The management of CKD in women of reproductive age requires a multifaceted approach. Early referral to nephrology, multidisciplinary collaboration, and tailored clinical guidance are essential to optimize the care and outcomes of this population. By recognizing the importance of preconception counseling, addressing modifiable risk factors, and enhancing awareness amongst healthcare providers, we can improve maternal and fetal health outcomes and reduce the progression of CKD. Further efforts to implement specific recommendations in this population are needed to ensure equitable and effective care for women affected by CKD.

## Data Availability

No datasets were generated or analysed during the current study.
